# Special Issue: Neural Bases of Cognitive Processing

**DOI:** 10.3390/brainsci11101286

**Published:** 2021-09-28

**Authors:** Francesco Di Russo, Stefania Lucia

**Affiliations:** 1 Department of Movement, Human and Health Sciences, University of Rome “Foro Italico”, 00135 Rome, Italy; s.lucia@studenti.uniroma4.it; 2 Santa Lucia Foundation IRCCS, 00179 Rome, Italy

The main aim of Cognitive Neuroscience is investigating how brain functions lead to mental processes and behavior. In this Special Issue of *Brain Sciences*, the articles have the goal to achieve a more sophisticated level of understanding of the neural basis of cognition, showing experimental techniques for studying functional interactions within the brain and models to better understand the basic component of human cognition, such as awareness, perception, attention, reasoning, language, decision making, memory, action control and executive functions, in healthy people and in patients with brain damage. This Special Issue will consider the mental processes through which we are aware, think, feel, act, learn, remember, and anticipate future events.

Devos et al. [1] take cognitive workload in consideration as a measure of physical and mental effort allocation to a task to provide information regarding possible cognitive decline, since, despite the power and flexibility, there are limits to a brain’s capacity. The purpose of this study is to establish the test–retest reliability of the National Aeronautics and Space Administration Task Load Index (NASA-TLX) and Index of Cognitive Activities (ICA), using pupillometry. The convergent validity of these measures against event-related potential (ERP) measures was also investigated. The results showed that pupillary response, transformed into ICA, provides a good measure of real-time cognitive workload in older adults with and without cognitive impairment. The NASA-TLX also offer an improved reliability in the assessment of cognitive workload in older adults. Moderate correlations were found between these two measures and the ERP P3 component.

Additionally, Koo et al. [2] use pupillometry to understand the capacity of working memory using, instead of a visual task, an auditory one. Experiment 1 examined the recall performance of spoken words in stationary noise and its correlation with Reading Span (RS) scores. Experiment 2 measured the recall performance of spoken words in both silent and four-talker babble conditions and evaluated its correlation with RS scores and pupillary measurements were taken. The results show that the noise did not appear to affect the free reenactment performance of normal-hearing adults, but other variables (serial position and RS group) had a significant effect on performance. This study explores the possibility of clarifying the neural basis of cognitive processing in auditory tasks for understanding of how listeners respond to a subsequent recall task in comparison with previously used methods.

Another study that uses free recall to investigate the neural basis of the brain networks associated with memory is the study by Neri et al. [3], which proposes an ecological task combined with fMRI acquisition to investigate the neural correlates of episodic memory. In particular, the authors concentrate on the analysis of the posterior parietal cortex, including the areas involved in memory retrieval: the angular gyrus (AG), the precuneus, the retrosplenial cortex (RSN), the inferior parietal lobule (IPL) and the inferior parietal sulcus, in which the task was to listen to a brief story and then perform a classic immediate recall task. They were then subjected to fMRI for 20 min where they alternated the free recall task with a backward counting task. After the fMRI the participants were taken to another room, and they were asked to recall the passage in a delayed recall task. Results on episodic memory during free recall task during fMRI scanning session show that the RSN and IPL patterns of activation are connected to the free recall activity, with the left AG highly recruited during the effort to retrieve verbal information. In addition, higher memory performance is linked to a balanced coactivation of the language default mode and Precuneus networks and a strong involvement of the anterior part of the left AG.

Another study using fMRI is that of Seghezzi et al. [4] who tested the sensorimotor control theory, which aims to predict the sensory consequences of actions and is essential to realize motor programs and efficiently process incoming sensory stimuli. The authors focus on the sense of agency and using the phenomenon of the intentional link as an implicit measure of the agency’s experience, so that the time interval between voluntary actions and their effects are perceived as shorter than their actual duration. In this regard, fMRI scanning was used in healthy adult participants engaged in an auditory and visual temporal judgment task under two conditions, active and passive, to understand, at the neurofunctional level, the role of the supplementary motor area (SMA) as a supra-modal hub for the sense of agency. The active trials consisted of autonomously pressing a button in response to visual or auditory targets; while in the passive trial participants were instructed to stay still and the experimenter responded to targets. After pressing the button, an action-consequence of a lightbulb or a sound was triggered with a variable delay, and the participants had to evaluate the perceived time interval between pressing the button and the action-consequence; thus, evaluating the sense of agency connected to the intentional link. The results confirmed that the SMA activity, in the anterior portion (pre SMA), is linearly associated with the magnitude of the intentional binding effect. This evidence provides support to the view that the brain mechanisms that give rise to our sense of agency are closely motor, completing the circle of a conceptual validation of the sense of agency as a phenomenon anchored to the motor system operations. Moreover, it allows us to generalize the relationship between pre-SMA and sense of agency as supra-modal.

Our brain continuously integrates and collects different information from different sensors systems to create a coherent and unified perception of the world of salient events. Integration usually improve perception, and the literature supports the hypothesis that it can occur across various stages of stimulus processing, including both bottom–up and top–down control. However, there is no evidence of anticipatory multisensory integration that occurs in before the stimulus presentation. The work of Fiorini et al. [5] focuses on pre-stimulus ERP recorded in two unimodal passive sensory tasks; auditory (A) and visual (V), and in an audiovisual multimodal task (AV). The ERPs obtained in the two unimodal tasks were then summarized and compared with the AV condition in order to detect possible anticipatory multimodal effect due to cortical multisensory integration in modality-specific sensory areas. The focus of the analyses was on the preparatory sensory ERP components’ visual negativity (vN) for visual modality and auditory positivity (aP) for auditory modality. The results showed that the vN and aP components have significantly greater amplitude and an earlier onset when the stimuli are presented simultaneously than for the sum of the unimodal conditions. Therefore, a stronger and earlier sensory preparation would be indicating the occurrence of cross-modal interactions even before the stimulus presentation. These results suggest that given the inevitable multisensory nature of the real-life environment, our brains seem to prepare in advance for multiple sources of sensory information to facilitate the real perception of events.

Another study that focuses on anticipatory brain activity that allows people to prepare for an upcoming task, is the work of Bianco et al. [6]. This study uses a manual version of the Stroop test during EEG recordings to better understand the neural basis of anticipatory activities in a discriminative response task. Using ERP measures this study aims to investigate in particular the Bereitschaftspotential (BP) that precedes any voluntary movement and to prefrontal negativity (pN) linked to cognitive preparation. The results show that the Stroop effect on cognitive functions is not limited to the reactive stage of processing, but it also involves proactive pre-stimulus activity, with a larger BP for incongruent than for congruent trials, but not pN effects. This therefore shows that the premotor areas play a key role in a neural basis in the adequate preparation for resolution of cognitive conflicts.

The work of Maffei et al. [7] analyzed the temporal dynamics of the interaction between emotion and attentional processes, studying ERPs associated with perception of emotional faces, measuring the N170 component, coding face structures, configurations and emotions; the early posterior negativity (EPN) reflecting advanced processing of the emotionally salient face, and the late positive potential (LPP) associated with allocation of processing/working memory resources to the motivational importance of emotional stimuli. The study included two tasks with identical stimuli but with different instructions. In the task on covert emotions, participants were asked to attend to the central square independently of the surrounding faces, and to choose the color of the square as quickly and as accurately as possible by pressing one of the four corresponding buttons; in the task dealing with overt emotions, participants were asked to categorize the expression (happy, fearful, sad, or neutral) conveyed by the face, regardless of the color of the central square. The results show that in the early stage of the face-sensitive N170, amplitude was significantly modulated by emotion but, in contrast, voltage in this early epoch did not vary as a function of the overt-covert task demands and, importantly, the emotion enhancement took place independently of overt vs. covert processing, without indication of top–down voluntary attentional modulation. For the intermediate epoch containing the EPN, there was a main effect of task requirements, with greater voltage in the overt compared to covert emotion task, as well as the main effect of emotion, but no evidence that EPN was influenced by a voluntary top–down processing of attention modulation. Finally, in the epoch that includes LPP, there were emotion-modulated amplitudes as a function of task demands, with greater amplitude improvements for openly processed fearful and sad faces than happy faces and, furthermore, an increase in top–down attentional resources were found in the overt task. These results allow to better understand the attentional temporal dynamics in the processing of an emotion on a face, demonstrating that the role of voluntary attention it begins at an intermediate stage and fully modulates the response to the emotional content in the final stage of processing.

The study by Sarrias-Arrabal et al. [8] investigates the relationship between the modulation of spectral bands and cognitive impairment in patients with multiple sclerosis (MS) during an oddball task. There were 30 patients with relapsing-remitting MS and 30 healthy controls (HC) that participated. While the subjects performed the task, evoked and induced alpha and gamma bands were analyzed with an EEG by applying temporal spectral evolution (TSE). The behavioral results showed slower response times and lower accuracy in MS patients than in the controls. TSE analysis revealed a delay in latency and lower amplitude of both evoked and induced alpha activity in MS patients than controls. Regarding the gamma band, there were no differences between the groups. In summary, patients showed deficits in early sensory (evoked alpha activity) and cognitive processing (induced alpha activity at longer latencies), while the induced gamma band supported the hypothesis of its role in translation of attentional focus (induced activity) and did not show strong activity in this paradigm (visual oddball). This study showed that patients with MS manifest a cognitive impairment shown by the behavioral parameters, which may be partially due to alterations of the main evoked and induced spectral responses.

To investigate cognitive function in patients with genetic disorders, the study of Palmieri et al. [9] focuses on language, and on executive and memory functions in patients with spino-bulbar muscular atrophy (SBMA), a rare neuronopathy that belongs to the family of motor neuron diseases. The aim of this study was to investigate empathic disorders of these patients, in terms of neural responses. Eighteen patients with SBMA and 18 healthy controls were submitted to ERPs measures during an empathy task. Participants were first presented with a sentence describing a painful or a neutral context followed by a face with a painful or a neutral expression and had to discriminate the face expression. An explicit questionnaire related to dispositional empathy was also administered to all participants, who were screened using neuropsychological battery tests that did not reveal potential cognitive deficits. The results of the study revealed enhanced neural empathic responses in patients with SBMA disease, especially for the early empathic response (sharing of experience, i.e., the most affective component of empathy), when both cues of empathy (i.e., context and face) were painful. Differently from controls, patients also showed an empathic response to painful faces preceded by neutral contexts. Overall, these findings improve knowledge on cognitive empathy in SBMA patients who appear to have a more empathic attitude, at least from the perspective of psychophysiological responsiveness.

Overall, this Special Issue offers a novel view on the neural bases of cognitive processes, focusing on anticipatory preparation for multisensory integration and in the Stroop task, sense of agency in motor tasks, episodic and working memory, emotion, attention, and cognitive load in heathy participants. In addition, two studies describe the neural basis of cognitive decline in MS patients and for an increased empathic response in SBMA patients. The used neurophysiological methods were mainly EEG/ERP, fMRI and pupillometry. These studies, from their uniqueness, may offer ideas for researchers working in this field as they integrate cognitive processes together with their neural bases, using innovative tasks adapted to the various research objectives.
